# Bird impacts on ecological structure, composition and function in Arctic ponds

**DOI:** 10.1007/s00300-025-03426-1

**Published:** 2025-10-23

**Authors:** Thomas C. Jensen, Ann Kristin Schartau, Birger Skjelbred, Alexander Eiler, Maarten J. J. E. Loonen, Annelies J. Veraart

**Affiliations:** 1 Norwegian Institute for Nature Research—NINA-Oslo, Sognsveien 68, 0855 Oslo, Norway; 2 Norwegian Institute for Water Research, Økernveien 94, 0579 Oslo, Norway; 3 Department of Biosciences, University of Oslo, Blindern, P.O. box 1066, 0316 Oslo, Norway; 4 Arctic Centre, University of Groningen, Aweg 30, 9718 CW Groningen, The Netherlands; 5 Department of Ecology, Radboud Institute for Biological and Environmental Sciences, Radboud University, P.O. Box 9010, 6500 GL Nijmegen, The Netherlands

**Keywords:** Eutrophication, Methane, Phytoplankton, Water chemistry, Zooplankton

## Abstract

Small ponds and lakes and their surrounding riparian zone are ecological hot spots in the Arctic landscape. They are important for biogeochemical cycling and habitats for terrestrial and aquatic organisms. Arctic birds may have strong impact on these small aquatic ecosystems. In particular, the increasing populations of Arctic geese are strongly associated with ponds and can significantly influence the physico-chemical properties and ecological communities of these waterbodies. Birds supply nutrients via feces, grubbing and physical disturbance leading to increased erosion and run-off. We examined how the pond ecosystems, including phytoplankton and zooplankton communities respond to increased bird impact. Six ponds on Svalbard differentially impacted by birds were sampled in summer 2022. Bird-impacted ponds were characterized by higher nutrient concentrations, especially of phosphorus. They also had significantly higher phytoplankton biomass with a different phytoplankton community. Although mixotrophic species constituted a substantial part of the community in all ponds, we observed increased dominance of both cryptophytes and cyanobacteria at high bird impact. High bird impact was also associated with low metazoan zooplankton abundance and high ciliate abundance. Phytoplankton and metazoan zooplankton taxa richness was much lower in the most impacted pond compared to the other waterbodies. Bird impact did not significantly relate to diffusive CO_2_ and methane fluxes from the ponds. Thus, high bird impact was associated with hypereutrophic pond conditions characterized by decreased diversity and a likely stimulation of the microbial loop. The results may hint at some of the future changes in Arctic ecosystems in ponds increasingly influenced by birds.

**Supplementary Information:**

The online version contains supplementary material available at 10.1007/s00300-025-03426-1.

## Introduction

Migrating animals can transport carbon and nutrients across large distances and across major ecosystem boundaries stimulating productivity of the recipient ecosystem (Bauer and Hoye 2014). In the Arctic, the transport of nutrients by seabirds from the marine environment to breeding areas in the terrestrial environment may contribute to increased productivity of an otherwise low-productive ecosystem (Brimble et al. 2009; Luoto et al. 2019). This surplus of nutrients of marine origin may also contribute to increased nutrient loadings to the freshwaters either from runoff from the breeding territories or directly from birds along the fly corridors (Gonzalez-Bergonzoni et al. 2017). The increasing Arctic goose populations provide another example of migrating animals serving as vectors for increased nutrient input from the terrestrial environment to the generally oligotrophic Arctic lakes and ponds (Van Geest et al. 2007; Mariash et al. 2018). These lentic freshwaters and surrounding wetlands are important ecological hot spots creating an Arctic pondscape, i.e., a network of ponds and lakes and their surrounding terrestrial matrix (Hill et al. 2018) in the otherwise barren Arctic landscape. They are essential parts of Arctic ecosystems which regulate carbon, water, and energy fluxes, and support a variety of fauna and flora and thus provide important ecosystem services (Rautio et al. 2011; Buij et al. 2017; Lento et al. 2019; Blackburn-Desbiens et al. 2023).

Arctic geese have increased in numbers in many Arctic regions (Jefferies et al. 2006; Pedersen et al. 2013; Flemming et al. 2016). This is also the case on Svalbard where the two most common species, the pink-footed goose (*Anser brachyrhynchus*) and the barnacle goose (*Branta leucopsis*) have experienced a dramatic increase in populations during the last half of the previous century (Madsen et al. 2017). The increased goose populations are partly a consequence of improved breeding conditions due to increased temperatures and an extended growing season and partly result from changes in land-use and hunting practices at overwintering sites in Western Europe (Madsen et al. 1999; Fox et al. 2010). The growing population of breeding geese has also led to a range expansion of their breeding and grazing grounds within the archipelago (Jensen et al. 2008; Wisz et al. 2008), thereby also affecting an increased number of terrestrial and aquatic habitats and the interaction between these habitats. It also appears that the population of the small seabird little auk (*Alle alle*), the most common bird in Svalbard, has increased over the past decades, at least in some areas on the archipelago (Luoto et al. 2019). Thus, it seems that increasing bird populations have become an important factor affecting the freshwater ecosystems on Svalbard during the last decades. Previous studies have shown that the increasing bird impact may affect water chemistry, such as pH and conductivity (Keatley et al. 2009; Gonzalez-Bergonzoni et al. 2017) and lead to higher inputs of nutrients contributing to bird-mediated eutrophication of the Arctic freshwater environment (Milakovic et al. 2001; Van Geest et al. 2007; Côté et al. 2010; Mariash et al. 2018). In turn, this may lead to community shifts in the generally oligotrophic species-poor Arctic ponds and lakes by increasing species richness and changing species composition (Jensen et al. 2019; Wei et al. 2023).

Despite an increasing number of studies of bird impact on Arctic lakes and pond ecosystems important knowledge gaps on the issue remain. It is well-established that increasing bird impact in Arctic ponds leads to eutrophication (e.g., Hessen et al. 2017; Mariash et al. 2018). However, these studies focused on specific parts of the pond food web, including phytoplankton and zooplankton composition, while other aspects of ecosystem functioning remained unresolved. For example, eutrophication leads to elevated methane emissions in temperate ecosystems (Davidson et al. 2015; West et al. 2016), but it remains unclear if bird-mediated eutrophication in Arctic ponds has a similar effect. At higher trophic levels, microcrustacean species richness and community composition has been shown to respond to increasing bird impact (Jensen et al. 2019), but nothing is known about impacts on other zooplankton groups such as rotifers and protozoans. Furthermore, higher primary production spiked by the increased nutrient availability at higher bird impact could increase zooplankton abundance (Saros et al. 2023), but to our understanding no studies have investigated this so far.

Our aim was to study how increasing bird impact affects ecological structure, composition and function of Arctic ponds. We hypothesize that (H1) increasing bird impact will lead to eutrophication with increasing phytoplankton biomass and zooplankton abundance and shifts in species composition. We also hypothesize that (H2) bird-induced eutrophication will lead to increased methane emission, and that this may be partly offset by higher daytime CO_2_ uptake due to increased photosynthesis.

## Methods

### Study sites

**Fig. 1:**
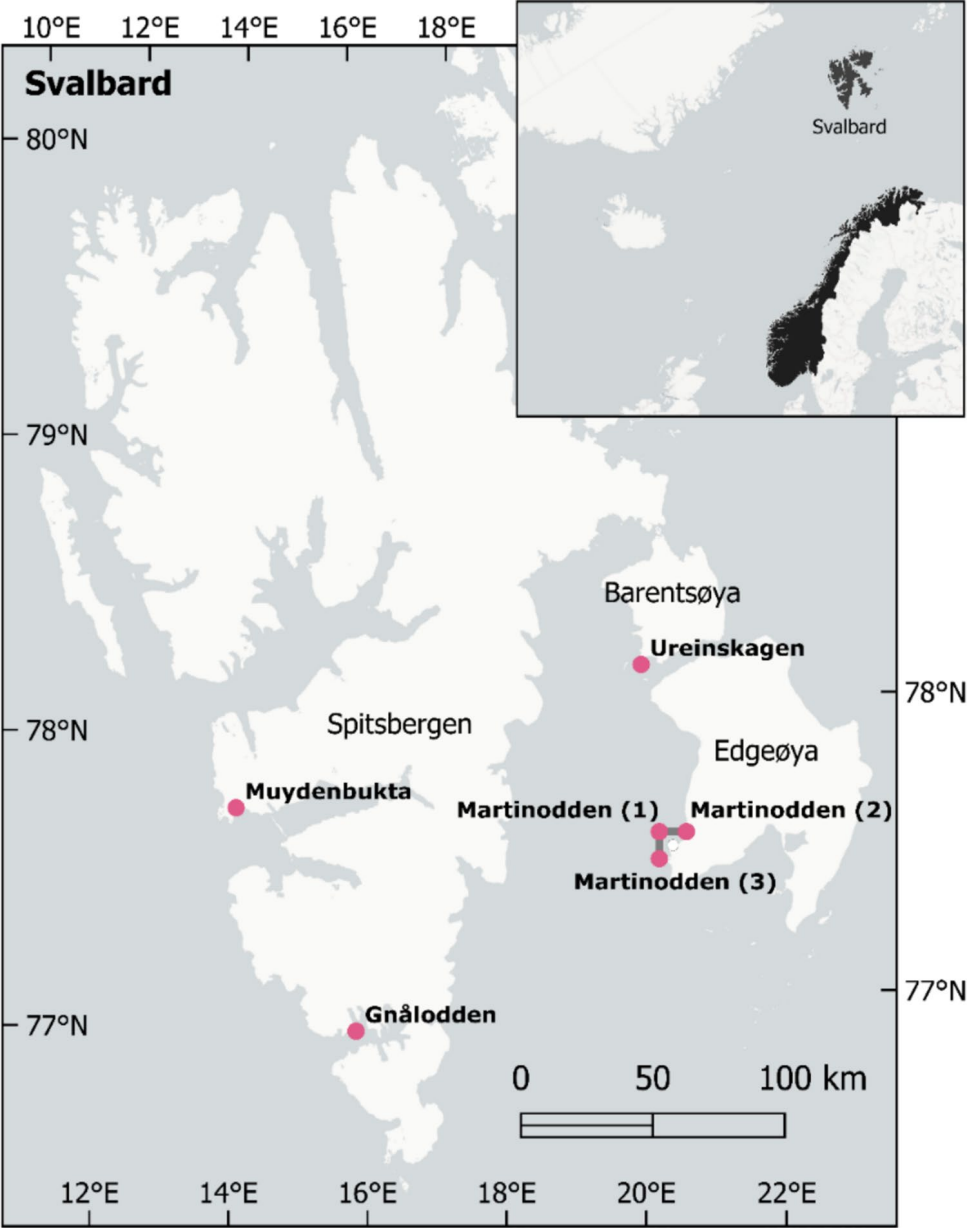
The location of the six studied ponds in Svalbard in 2022. The ponds sites were distributed as follows: Ureinskagen at Barentsøya (one pond), Martinodden at Edgeøya (three ponds) and Gnålodden and Muydenbukta at South-Western Spitsbergen (two ponds). None of the ponds had names from before. We named the ponds according to their geographical location. The three ponds at Martinodden were named Martinodden (1), Martinodden (2) and Martinodden (3)

A survey of six ponds at Barentsøya, Edgeøya and South-Western Spitsbergen, Svalbard (Fig. 1), which covered a gradient of goose and sea bird abundance, was carried out in July 2022 during the Scientific Expedition Edgeøya Spitsbergen. One of the study ponds was located on a small Island, Ureinskagen, south-west of Sundbukta, Barentsøya. Three other study ponds were located on Martinodden south of Russebukta, Edgeøya, and the last two ponds were located on Spitsbergen, respectively at Gnålodden in Hornsund and north of Van Muydenbukta in Bellsund. None of the ponds, had names from before. For practical reasons ponds were named according the geographical names mentioned above. The three ponds at Martinodden were named Martinodden (1), Martinodden (2) and Martinodden (3). This naming is used in the manuscript. However, in the graphic presentation of the results ponds are arranged in according to increasing bird impact.

The ponds were exposed to different levels of goose abundance. We counted the number of area-specific goose droppings, to quantify goose presence and abundance (range of 1–22 droppings/m^2^). In addition to high goose abundance, pond Gnålodden was located in front of a bird cliff and was therefore also exposed to seabirds.

The surface-area of the ponds varied between approximately 0.02 ha and 13.8 ha with maximum depths of ≤ 1 m (Table 1). None of the sites were directly under marine impact, although some of the ponds likely received sea spray. All sites were devoid of fish. Due to logistical challenges inherent to high Arctic fieldwork, pond Gnålodden was sampled using a trimmed down protocol.

**Table 1:** Major characteristics of the six Svalbard ponds

	Ureinskagen	Martinodden (1)	Martinodden (2)	Martinodden (3)	Gnålodden	Muydenbukta
Elevation (m a.s.l.)	5	15	18	13	2	13
Area (ha)	0.908	0.566	0.519	0.024	0.047	13.750
Nb goose droppings (/m2)	6.4	2.1	6.7	1.3	20.3	21.9
Goose droppins-to-pond-area ratio	7.1	3.8	12.9	56.6	429.9	1.6
Oxygen concentration (mg O2/l)	13.3	12.8	11.2	13.0	12.7	11.2
Oxygen saturation (%)	117.30	122.40	105.1	119.06	107.30	107.6
Conductivity (µS/cm)	472	976	110	201	895	140
pH	7.2	7.4	7.5	8.5	7.0	7.3
Tot-P (µg P/L)	2	3	4	58	564	13
TN (µg N/L)	280	360	380	620	1070	110
TOC (mg C/L)	5.8	9.6	9.8	14.2	9.3	2.4
methane flux (mg/m2/d)	166	1209	2582	3952		339
Pond NO3 (µg N/l)	0.0	2.1	5.9	42.4		3.6
Pond NH4 (µg N/l)	65.2	50.0	29.5	113.0		63.6
Pond PO4 (µg P/l)	3.2	1.6	1.6	5.8		1.6
Pond Al (µg/l)	0.0	8.2	0.0	0.0	11.2	0.0
Pond Ca (mg/l)	26.6	21.1	4.1	6.1	50.0	19.6
Pond Cl (mg/l)	45.6	175.3	21.4	16.3	83.1	5.8
Pond Fe (µg/l)	82	390	107	302	849	113
Pond K (mg/l)	1.7	3.5	0.9	1.6	6.6	0.2
Pond Mg (mg/l)	13.3	12.1	1.6	1.9	7.3	4.0
Pond Mn (µg/l)	0.2	19.7	0.0	34.7	7.0	0.0
Pond (Na mg/l)	44.3	107.2	13.0	33.8	35.8	3.7
Pond S (mg/l)	25.7	9.8	0.5	3.9	4.1	1.4
Pond Si (mg/l)	0.2	0.3	0.2	0.9	0.0	0.4
Pond Zn (µg/l)	0.0	5.7	7.5	10.7	5.8	18.2
Pore NO3 (µg N/l)	1203.4	819.4	219.1	6.3		2.1
Pore NH4 (µg N/l)	2810.9	6367.9	3059.5	1236.7		613.9
Pore PO4 (µg P/l)	47.6	11.6	406.8	230.3		4.2
Pore Al (µg/l)	55.2	60.3	25.3	321.7		0.0
Pore Ca (mg/l)	69.6	227.5	53.1	16.9		55.7
Pore Cl (mg/l)	106.9	1744.1	215.2	44.4		34.8
Pore Fe (µg/l)	1257.3	309.7	8422.5	12,143.9		879.0
Pore K (mg/l)	5.7	23.4	16.3	1.9		1.6
Pore Mg (mg/l)	34.3	148.4	26.3	5.7		14.3
Pore Mn (µg/l)	460.3	1240.1	812.2	367.0		230.4
Pore (Na mg/l)	123.3	811.7	140.1	68.6		26.3
Pore S (mg/l)	90.8	228.4	24.3	1.4		4.9
Pore Si (mg/l)	10.2	6.0	5.4	8.1		4.3
Pore Zn (µg/l)	36.9	85.1	67.6	62.4		25.8

### Sampling and analysis

From each of the six ponds a single 5-L composite water sample was taken from approximately 0.2 m below the surface for subsampling and later analysis of water chemistry and phytoplankton. All sampling equipment was first rinsed with a 1% bleach solution and then decontaminated with ethanol (70%) prior to sampling. Temperature, oxygen content, conductivity and pH were measured with a HI98494 multiparameter Bluetooth portable pH/EC/OPDO meter (Hanna Instruments) on site.

Additional surface water samples and porewater samples were collected in ponds 1–4 and 6, to determine dissolved nutrient and trace element concentrations. To this end, porewater samples were collected at a representative site of the pond, around 3–4 m from the bank, using ceramic porewater samplers at a depth of ≈ 5–10 cm in the sediment. By creating a vacuum, two samples of filtered porewater were collected, one of which was frozen at − 20 °C until analysis, and the other was acidified using 0.2 ml 35% HNO_3_ and stored cool. In addition, two 20 ml surface water samples were collected from the composite water sample, one of these samples was syringe-filtered (0.22 µm) immediately in the field, while the other was acidified and stored as described above.

Total phosphorus and dissolved phosphorus were measured on an auto-analyser as phosphate after wet oxidation with peroxodisulfate. Total nitrogen was measured as nitrogen monoxide by chemiluminescence using a TNM-1 unit attached to the Shimadzu TOC-VWP analyser (Shimadzu Corporation, Japan). Total organic carbon (TOC) was obtained by analysis on a Shimadzu TOC-L with sample changer ASI-L (Shimadzu Corporation, Japan). Analysis of total phosphorus, total nitrogen and TOC were conducted at Department of Biosciences, University of Oslo, Norway. Dissolved NO_3_^−^, NH_4_^+^, PO_4_^3−^ were measured colorimetrically in filtered samples using a SAN^plus^ autoanalyzer (Skalar Analytical, Breda, the Netherlands). Concentrations of Al, Ca, Cl, Fe, K, Mg, Mn, Na, S, Si, and Zn were measured in the acidified samples using inductively coupled plasma optical emission spectrometry (ICP-OES, Thermo Fischer Scientific, Bremen, Germany). Analysis of dissolved NO_3_^−^, NH_4_^+^, PO_4_^3−^ and trace elements were conducted at the Radboud Faculty of Science General Instrumentation Facility, Nijmegen, the Netherlands.

For quantification of phytoplankton and ciliate abundance and species composition, a 100 ml subsample from the composite water sample was fixed with acid Lugol’s solution where 0.5 ml Lugol’s solution were added to the phytoplankton sample. The phytoplankton samples were kept cold (5–10 °C) and dark until analysis.

We took two zooplankton samples from each of the ponds; one semi-quantitative sample was taken with a plankton net (diameter 30 cm, mesh size 45 µm) and one quantitative sample (20 L) taken with a bucket and filtered through the plankton net. Zooplankton samples were fixed with acid Lugol’s solution where 1.0 ml Lugol’s solution were added to the zooplankton sample. They were stored dark until analysis.

Goose abundance were quantified by counting droppings in 0.25 m^2^ quadrants along three, 6-m transects from the shoreline, as described in detail in Jensen et al. (2019). The impact of the goose-mediated input of nutrients and ions partly depend on pond size. For instance, the nutrient and ionic concentrations of a larger pond may be lower compared to a smaller pond with similar goose dropping densities on the shores, due to dilution from the larger water volume. To compensate for variable pond size when considering goose impact, we calculated the droppings-to-pond-area ratio as a proxy for goose impact.

In ponds Ureinskagen, Martinodden and Muydenbukta, diffusive CO_2_ and methane flux between water surface and air was measured in brief time series. A transparent, floating flux chamber (ø 29.1 cm, height 21.4 cm) was connected to a Li-7810 trace gas analyser using air-tight Tygon tubing (ø 4 mm, L 20–40 m). Fluxes were measured in fourfold, each measurement lasting 3 to 7 min, until a linear change was observed without disturbance by methane ebullition events. After each measurement, the chamber was vented until atmospheric CO_2_ and CH_4_ concentrations were reached. Average wind speed and air temperature were recorded during each measurement and used to calculate water–air fluxes according to Zhao et al. (2018), using the *gas fluxes* package in R. Flux measurements with obvious ebullition events were not used for diffusive flux calculations.

Phytoplankton and ciliates were quantified using inverted microscopy, following standard methods (NS-EN-15204 2006, EN-16695 2015). The taxonomical nomenclature of phytoplankton and ciliates followed Guiry and Guiry (2024) and Foisssner and Berger (1996), respectively. Counting units were generally resolved to species level except for small, naked flagellates which are impossible to identify by light microscopy. The number of counting units varied from 394 to 2106 per sample, with a median of 784 units. Phytoplankton counts were converted to bio-volumes by multiplying cell abundances with average cell volumes estimated from measured linear dimensions and geometrical formulae. Each sample was examined at different magnifications to also cover large-celled species with low abundance, but high contribution to bio-volume.

Zooplankton samples were identified and counted with the use of a dissecting microscope. All crustaceans were identified to species, while rotifers in most cases were identified to genus. For identification we followed Flößner (1972, 2000), Kiefer (1978) Pontin (1978), Einsle (1993, 1996) and Bledzki and Rybak (2016).

Correlation analysis (Pearson’s correlation, r) was used to evaluate relationships between bird impact (droppings-to-pond-area ratio) and the investigated variables. Normal distributions were checked by visual inspection of the residuals. Non-normal distributed variables were transformed (log_10_(x + 1)).

## Results

**Fig. 2:**
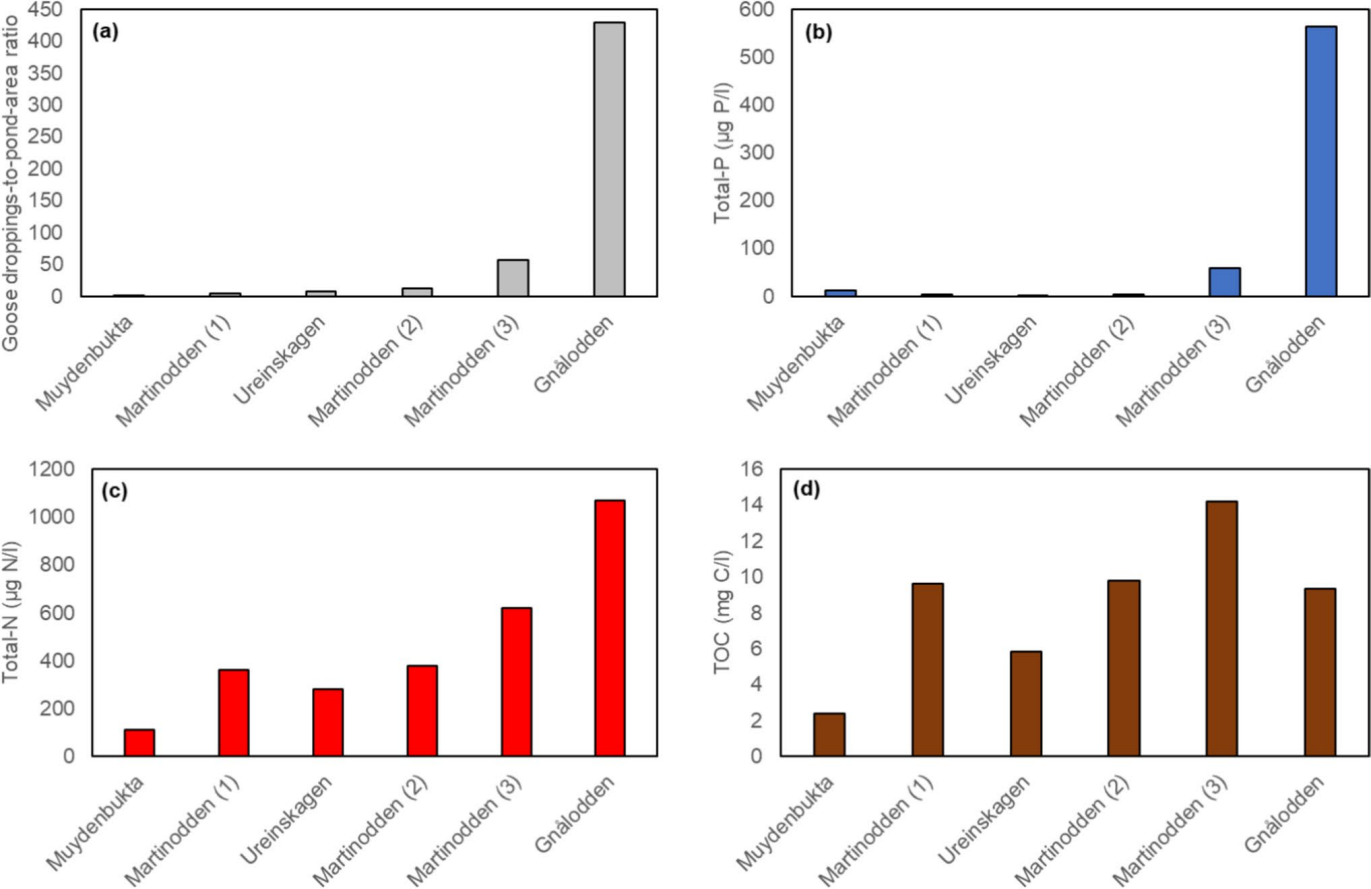
Goose droppings-to-pond-area ratio (**a**), concentrations of total phosphorus (**b**), total nitrogen (**c**) and total organic carbon (**d**) in pond water. Ponds are arranged according to increasing bird impact from left to right, i.e., increasing goose droppings-to-pond-area ratio. Additionally, pond Gnålodden was in front of a bird cliff and thus received additional nutrient input from seabirds

**Fig. 3:**
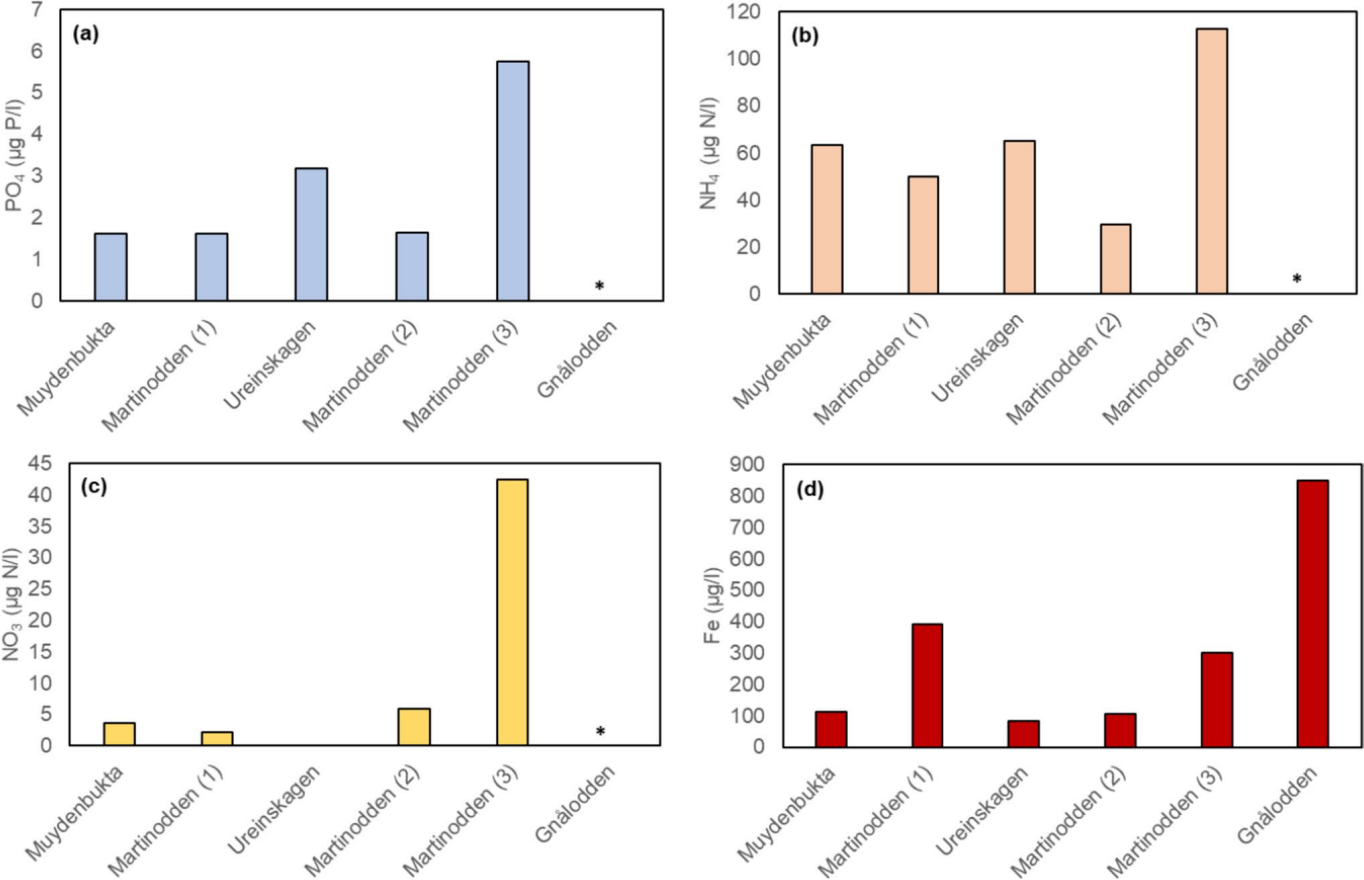
Concentrations of PO_4_-P (**a**), NH_4_-N (**b**), NO_3_-N (**c**) and Fe (**d**) in pond water. Missing values for PO_4_-P, NH_4_-N, NO_3_-N (“*”) in pond Gnålodden. Ponds are arranged according to increasing bird impact from left to right, i.e., increasing goose droppings-to-pond-area ratio. Additionally, pond Gnålodden was in front of a bird cliff and thus received additional nutrient input from seabirds

The ponds were located between 2 and 18 m above sea level, and they were all smaller than 1 ha, except pond Muydenbukta with an area of approximately 14 ha (Table 1). All ponds were relatively shallow (max. depth < 1 m) and therefore freeze to the bottom in winter and are thus devoid of fish. The ponds were oversaturated with oxygen at the time of measurement (Table 1). The concentrations of ions were moderate to high; conductivity ranged from 110 µS/cm to 976 µS/cm (median 337). The pH was between 7.0 and 8.5 (median 7.4). Relatively high Cl concentrations in the surface water and pore water of pond Martinodden (1) could indicate some influence of sea water (Table 1). The ponds Ureinskagen, Martinodden (1), Martinodden (2) and Muydenbukta had lowest bird influence; having droppings-to-pond-area ratios between 2 and 13. Pond Martinodden (3) had intermediate bird influence, at a droppings-to-pond-area ratio of 57; while pond Gnålodden had a high bird influence with a droppings-to-pond-area ratio of 430 (Table 1, Fig. 2a). Additionally, this pond was impacted by seabirds due to the location below a bird cliff. The bird impact was reflected in the nutrient concentrations in the water. Thus, in ponds Ureinskagen, Martinodden (1), Martinodden (2) and Muydenbukta total phosphorus was low, while pond Martinodden (3) and especially pond Gnålodden had much higher concentrations (Fig. 2b). Overall, bird impact (log10(Goose droppings-to-pond-area ratio + 1) was significantly correlated with total phosphorus concentration (log_10_(total phosphorus + 1), r = 0.89, *p* = 0.018). For total nitrogen the pattern was similar, but the increase in ponds Martinodden (3) and pond Gnålodden was not as steep as for total phosphorus (Fig. 2c). Overall, bird impact was significantly correlated with total nitrogen concentration (*r* = 0.98, *p* < 0.001). The concentration of total organic carbon did not reflect the bird impact (Fig. 2d, *r* = 0.57, *p* > 0.239). Values for inorganic phosphorus and nitrogen were missing for pond Gnålodden but concentrations of PO_4_-P, NH_4_-N, NO_3_-N where higher in pond Martinodden (3) than in the four ponds of lower bird impact (Fig. 3a–c). However, bird impact was not significantly correlated with inorganic nutrients (PO_4_: *r* = 0.84, *p* = 0.073; NH_4_: *r* = 0.59, *p* = 298; log_10_(NO_3_ + 1): *r* = 0.73, *p* = 0.166). The concentrations of Fe, Ca, and K (Table 1, Fig. 3d) were also highest in pond Gnålodden, the pond with the highest bird impact, but bird impact was not significantly correlated with any of these ions (Fe: *r* = 0.80, *p* = 0.054; Ca: (*r* = 0.49, *p* = 0.319; K: *r* = 0.74, *p* = 0.093). High concentrations of Fe, Ca and K may be due to input from seabirds, as pond Martinodden (3), with relatively high goose impact, did not have elevated concentrations of these ions. Analysis of pore water were missing for pond Gnålodden. However, bird impact did not have any noticeable impact on the measured inorganic nutrients and Fe, Ca, and K in pond Martinodden (3) of intermediate bird impact (Table 1).

**Fig. 4:**
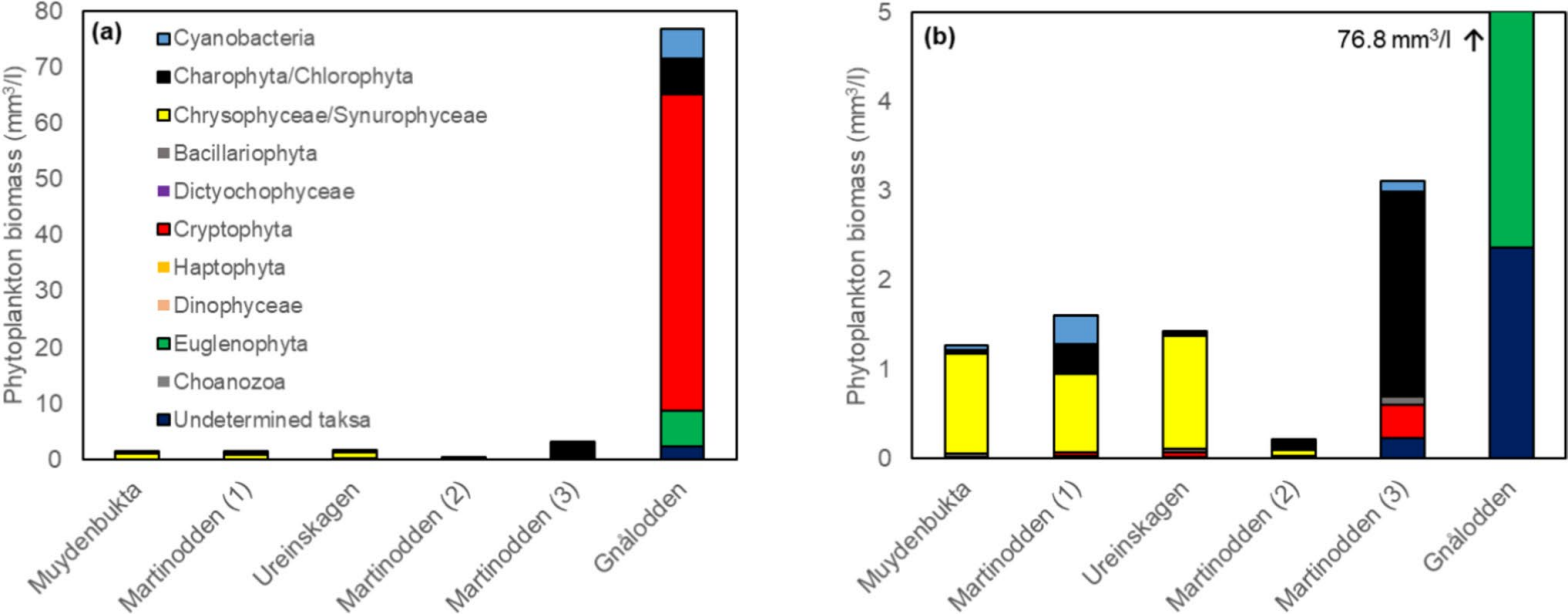
Phytoplankton biomass (**a**), phytoplankton biomass enlarged to ease the visibility of the different phytoplankton groups in the five ponds with lowest phytoplankton biomass (**b**). Ponds are arranged according to increasing bird impact from left to right, i.e., increasing goose droppings-to-pond-area ratio. Additionally, pond Gnålodden was in front of a bird cliff and thus received additional nutrient input from seabirds

The phytoplankton biomass remained low (< 1.6 mm^3^/l) in the four ponds of low bird impact and with low nutrient concentrations (Fig. 4a–b). Pond Martinodden (3), with intermediate bird impact and higher nutrient concentrations also had higher phytoplankton biomass. Pond Gnålodden, with concomitant impact of geese and seabirds and with the highest nutrient concentrations, had exceptionally high phytoplankton biomass of 77 mm^3^/l. Overall, bird impact was significantly correlated with phytoplankton biomass (log_10_(phytoplankton biomass + 1), *r* = 0.85, *p* = 0.032). In addition ponds Martinodden (3) and Gnålodden, also had a clearly different phytoplankton community composition compared to the four ponds of low bird impact (Fig. 4a–b). In ponds Ureinskagen, Martinodden (1) and Muydenbukta the phytoplankton community was dominated by Chrysophytes, mainly *Uroglenopsis americana*, *Chromulina* spp. *Ochromonas* spp. Species from the Synurophyceae genus *Mallomonas* were also observed in ponds Ureinskagen and Martinodden (2). In ponds Martinodden (2) and Martinodden (3) Chlorophytes like *Chlamydomonas* and green spherical cells dominated the phytoplankton community. Several taxa of Charophytes were observed in pond Martinodden (2). Chlorophytes and Cyanophyceae were sub dominant groups in ponds Martinodden (1) and Gnålodden. The blue-green mixotrophic Cryptophyte *Chroomonas* sp. dominated pond Gnålodden along with Euglenophytes, Chlorophytes and Cyanophyceae as sub-dominating groups. While the biomass of species capable of mixotrophy increased at high bird impact they constituted a substantial part of the community in all the ponds. Furthermore, at low bird impact, mixotrophs were dominated by Chrysophytes (Table S1), while Cryptophytes dominated at intermediate and high bird impact (Table S1). The biomass of heterotrophic flagellates also increased at intermediate and especially at high bird impact (Table S1).

**Fig. 5:**
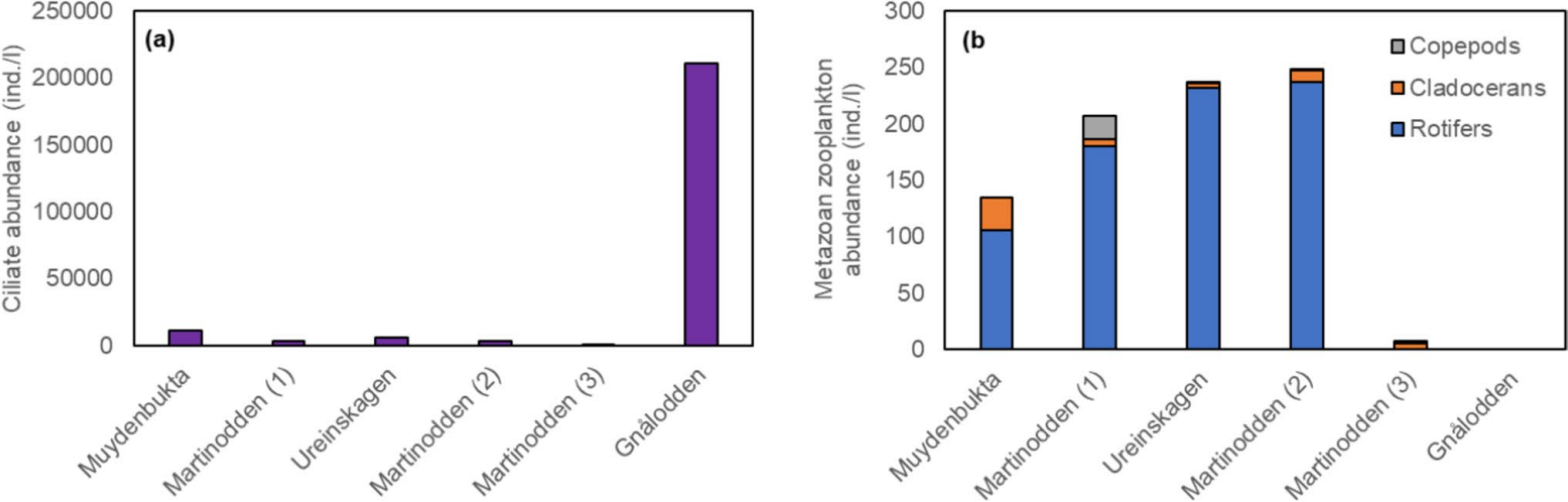
Abundance of ciliates (**a**) and metazoan zooplankton; rotifers, cladocerans and copepods (**b**). Ponds are arranged according to increasing bird impact from left to right, i.e., increasing goose droppings-to-pond-area ratio. Additionally, pond Gnålodden was in front of a bird cliff and thus received additional nutrient input from seabirds

In the four ponds of lowest bird impact, as well as in pond Martinodden (3) with the next highest bird impact, the abundance of ciliates was low and varied between 250 and 11,100 ind./l (Fig. 5a). In pond Gnålodden, with the highest bird impact, the abundance of ciliates reached extreme densities of 211,000 ind./l. Overall, bird impact was not significantly correlated with abundance of ciliates (log_10_(ciliate abundance + 1), *r* = 0.35, *p* = 0.492). Metazoan zooplankton abundance increased slightly from pond Muydenbukta to pond Martinodden (2) along the weak increase in bird impact, largely due to increasing rotifer abundance (Fig. 5b). In pond Martinodden (3), with the next highest bird impact, metazoan zooplankton abundance was very low, because of an almost complete disappearance of rotifers. However, copepods and cladocerans were still present in this pond, although in lower abundance than in ponds Ureinskagen and Martinodden (1) with the lowest droppings-to-pond-area ratios. Zooplankton samples were not taken in pond Gnålodden, to avoid clogging of the zooplankton net with phytoplankton mats and other organic material of very slimy texture in this pond, which would have impaired zooplankton sampling during the remaining part of the sampling campaign. The microcrustaceans were visible by the naked eye in all the other sampled ponds. Visual inspection showed that microcrustaceans were absent in pond Gnålodden. Bird impact was not significantly correlated with metazoan zooplankton abundance (*r* = − 0.54, *p* = 0.344).

**Fig. 6:**
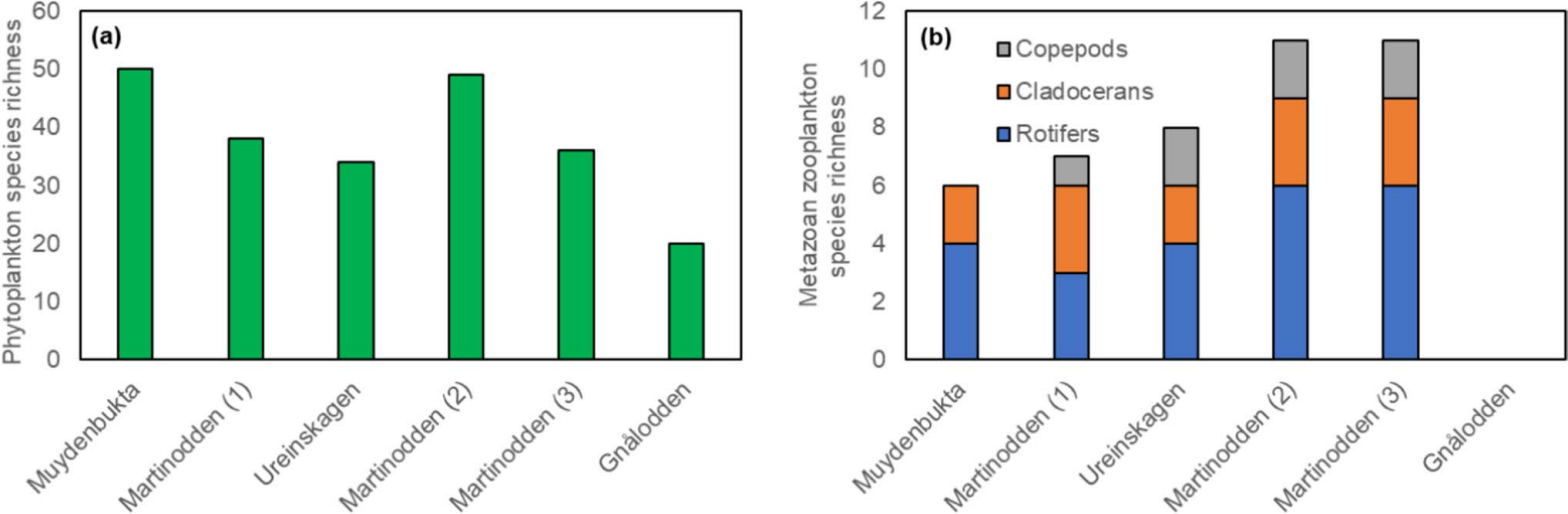
Species richness of phytoplankton (**a**) and metazoan zooplankton (rotifers, cladocerans and copepods; (**b**). Ponds are arranged according to increasing bird impact from left to right, i.e., increasing goose droppings-to-pond-area ratio. Additionally, pond Gnålodden was in front of a bird cliff and thus received additional nutrient input from seabirds

Phytoplankton species richness varied widely between ponds, with pond Gnålodden having the lowest richness (Fig. 6a). Bird impact was not significantly correlated with phytoplankton species richness (*r* = − 0.79, *p* = 0.059). Metazoan zooplankton species richness increased with rising bird impact from pond Muydenbukta to ponds Martinodden (2) and Martinodden (3) (Fig. 6b). This was primarily linked to the increase in the species richness of rotifers. However, in pond Gnålodden the metazoan zooplankton species completely disappeared except for one rotifer species recorded in the phytoplankton sample (not shown in Fig. 6b). Bird impact was significantly correlated with metazoan zooplankton species richness (*r* = 94, *p* = 0.019). Five microcrustaceans (*Daphnia pulex, Chydorus sphaericus, Macrothrix hirsuticornis, Eurytemora raboti and Diacyclops crassicaudis*) and 10 rotifers (*Brachionus* sp., *Colurellqa* sp., *Polyarthra* sp., *Notholca* sp., *Kellicotia longispina*, *Synchaete* sp., *Trichocerca* sp., Lecane sp., *Keratella quadrata* and Rotatoria sp.) were recorded in the ponds. *Daphnia pulex*, *Chydurus sphaericus* and *Eurytemora raboti* were the most common microcrustaceans and *Polyarthra* sp. was by far the most common rotifer. Arctic tadpole shrimp, *Lepidurus arcticus*, was present in all the ponds except pond Gnålodden (data not shown).

**Fig. 7:**
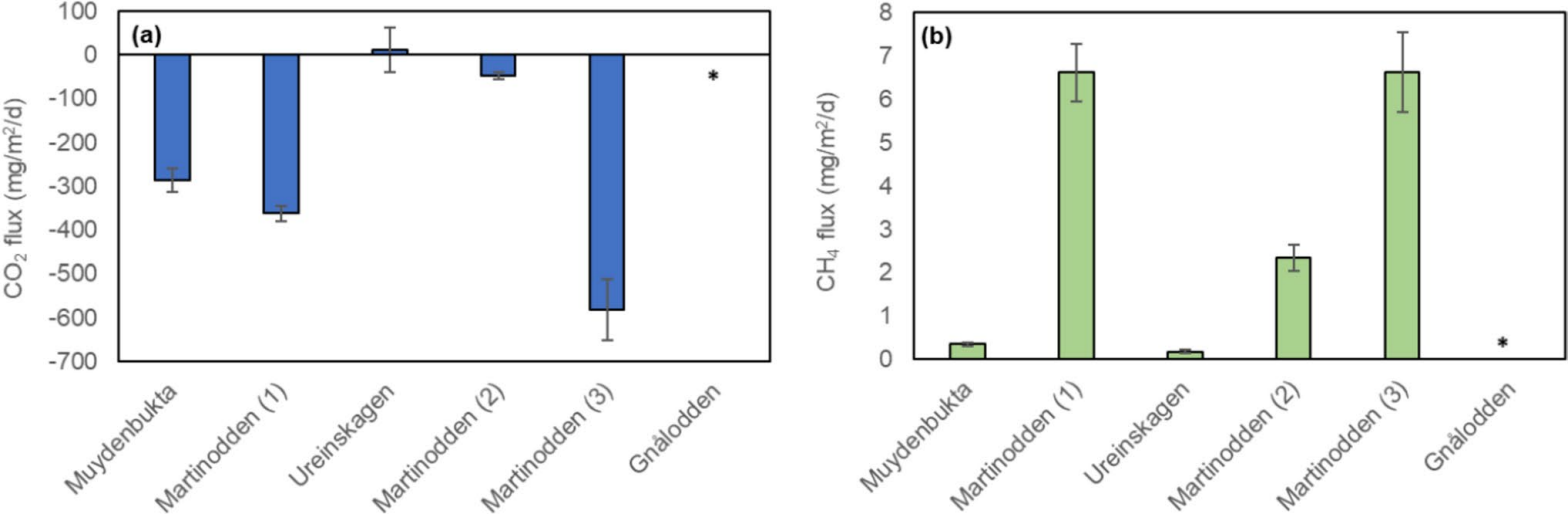
Diffusive water–air CO_2_- (**a**) and CH_4_-flux (**b**) measured in fourfold at each pond (mean ± standard error). Missing values (“*”) in pond Gnålodden. Ponds are arranged according to increasing bird impact from left to right, i.e., increasing goose droppings-to-pond-area ratio. Additionally, pond Gnålodden was in front of a bird cliff and thus received additional nutrient input from seabirds

CO_2_ flux’ were negative in all ponds ranging from −581 mg/m^2^/day, except pond Ureinskagen with a slightly positive emission of 11.4 mg/m^2^/day (Fig. 7a). Thus, ponds Martinodden (1), Martinodden (2), Martinodden (3) and pond Muydenbukta had a net CO_2_ uptake, while pond Ureinskagen had small net release. Bird impact was not significantly correlated with CO_2_ flux (*r* = − 0.38, *p* = 0.523). Although the number of observations was low, the CO_2_ flux seemed to be inversely correlated with total phosphorus, phytoplankton biomass and pH, indicating that the high CO_2_ uptake in pond Martinodden (3) reflected the high photosynthetic activity in this pond. Diffusive methane emissions were detectable but low in all ponds, ranging from 0.18 till 6.6 mg/m^2^/day (Fig. 7b). There was no clear relation between bird impact and diffusive methane emission (*r* = 0.51, *p* = 0.384), but methane emission tended to be higher at higher water column TOC and water column Fe concentrations. Moreover, part of the CH_4_ emission was in the form of bubbles, which were observed in ponds Martinodden (3) and Muydenbukta, but ebullition was not quantified due to time constraints.

## Discussion

Our study, contrasting Arctic ponds of different bird impact, shows increasing nutrient concentrations stimulating phytoplankton biomass at relatively high bird impact using the droppings-to-pond-area ratio as a proxy for bird abundance. This bird-mediated eutrophication was even stronger under the combined effects of geese and seabirds where nutrients reached very high concentrations spiking a particularly high phytoplankton biomass. The high bird impact was associated with clear community shifts of phytoplankton including an increase in the biomass of mixotrophic and heterotrophic species, a higher share of cyanobacteria and lower phytoplankton species richness at the highest bird impact. Bird impact seemed to have a strong effect on zooplankton. Ponds with low bird-mediated eutrophication were characterized by high metazoan zooplankton abundance, dominated by rotifers, and low abundances of ciliates. High bird presence was associated with low metazoan zooplankton abundance in which rotifers were virtually absent. At the highest bird impact, crustacean zooplankton was absent and displaced by ciliates in extreme abundances. There was no clear relation between diffusive methane flux and bird-pressure, but methane emission tended to be higher at higher concentrations of TOC and Fe in the water column.

Our results show that high bird impact is associated with elevated nutrient concentrations and higher phytoplankton biomass, which is in accordance with previous studies demonstrating bird-mediated eutrophication in Arctic ponds (Keatley et al. 2009; Jensen et al. 2019; Nagar et al. 2022). The concentration of total phosphorus in pond Martinodden (3), the pond with the second highest bird impact judged by the goose droppings-to-pond area ratio, was in the upper range reported by Hessen et al. (2017). Concerning trophic status (Vollenweider and Kerekes 1982), the ponds of relatively low bird influence (ponds Ureinskagen, Martinodden (1), Martinodden (2) and Muydenbukta) were in the oligotrophic range or on the border to mesotrophic regarding total phosphorus. Pond Martinodden (3) was in the eutrophic range and pond Gnålodden, impacted by seabirds in addition to geese, was in the hypereutrophic range. The high concentrations of Fe in pond Gnålodden is likely due to seabird impact as pond Martinodden (4) with high goose impact and no or insignificant seabird impact did not have elevated Fe concentrations. Furthermore, other studies also indicated increased Fe concentrations in seabird-impacted ponds (Mallory et al. 2006; Duda et al. 2018, 2021), while ponds under higher goose impact did not have elevated Fe concentrations (Mallory et al. 2006; Mariash et al. 2018). Geese are mainly herbivorous (e.g., Jeffries et al. 2006), whereas seabirds eat fish and therefore biomagnify Fe (Wing et al. 2014). Evidence suggests that Fe plays an underappreciated role in limiting primary production in high-latitude freshwaters (Vrede and Tranvik 2006) with seabirds providing an important source of Fe in Arctic ponds limiting primary production.

As pond Martinodden (3) with elevated goose impact did not have higher pore water concentrations of nutrients or other ions, the pore water geochemistry did not seem to track the bird impact in this pond. Due to the lack of measurement of sediment geochemistry, we do not know if pond Gnålodden, with the highest bird impact, had a eutrophication imprint on the pore water geochemistry as has been shown previously for Arctic ponds with seabird impact (Duda et al. 2018). However, due to the hypertrophic conditions of pond Gnålodden, this seems likely.

In accordance with our results there are indications of elevated primary production in bird-impacted Arctic ponds (Jensen et al. 2019; Duda et al. 2021). However, bird-mediated nutrient enrichment may not necessarily lead to higher standing stock of algae in these Arctic water bodies (Van Geest et al. 2007; Coté et al. 2010; MacDonald et al. 2014, 2015). In the absence of efficient top-down control from fish, high densities of zooplankton grazers (notably *Daphnia*) may control algal biomass (Van Geest et al. 2007; van der Waal and Hessen 2009). For shallow, Arctic ponds, however, it is likely that a major portion of released nutrients from bird faeces is taken up by benthic autotrophs, but these effects are scarcely studied. The marked changes in phytoplankton composition between high and low bird impact are in accordance with previous studies (e.g., Keatley et al. 2011; Jensen et al. 2019). We found a high fraction of Charophyta/Chlorophyta with cryptophytes as subdominant taxa at intermediate goose impact, and at the highest bird impact cryptophytes dominated and the biomass of cyanobacteria were also higher than in ponds with low bird impact. Other studies have shown that mixotrophic cryptophytes increase with eutrophication (Saad et al. 2016), whereas Mariash et al. (2019) found cyanobacteria to be dominant or subdominant in enrichment experiments of Arctic pond water with goose droppings. Climate change is causing increased occurrence of cyanobacteria in temperate and high-latitude lakes (Pick et al. 2016). This may have important consequences for ecosystem functioning in lentic systems for example through blooms, which may stimulate anoxia upon decay. In temperate regions the increased incidences of cyanobacterial blooms due to climate change may also be associated with toxin formation, thus threatening the ecosystem services provided by surface waters (Huisman et al. 2018). Likewise, higher temperatures in the Arctic may also be associated with higher incidence of toxin producing cyanobacteria (Kleinteich et al. 2013). Increasing impact from growing bird populations in the Arctic may interact with climate change to increase the occurrence of cyanobacteria at the regional or local scale.

In contrast to our hypothesis of increasing zooplankton abundance with increased bird impact, our results indicate that the metazoan zooplankton abundance peaked at low to moderate bird-influenced nutrient enrichment. Rotifers were virtually absent in goose-impacted pond Martinodden (3), and crustacean zooplankton was not observed in pond Gnålodden, which was impacted by both geese and seabirds. Also, *Lepidurus arcticus* was absent from pond Gnålodden. In contrast, protozoan zooplankton, i.e., ciliates, thrived in this pond. Likely, hypertrophic conditions in this pond was associated with a deteriorated environment not supportive of a range of organisms found in the other ponds. High oxygen saturation measured at daytime during the field campaign indicated that oxygen depletion was not the reason for environmental degradation. Oxygen saturation was likely also high during night because of photosynthesis during the polar night. Probably, pond Gnålodden had extraordinarily high ammonium concentrations due to very high degradation of organic material in addition to direct input of ammonia reflecting the high content of ureic acids in seabird feces. Previously extremely high levels of ammonia were found in ponds affected by gulls (Loder et al. 1996). Likely, ammonia reached levels toxic to metazoan zooplankton (Yang et al. 2017), as well as other invertebrates, e.g., *L. arcticus.* Unfortunately, we have no measurements of ammonia from pond Gnålodden to support this. Ciliates tolerate very high concentrations of ammonia (e.g., Klimek et al. 2012), and with the disappearance of larger potential predators and competitors, ciliates apparently thrived in pond Gnålodden. Deteriorated environmental conditions with high ammonia concentrations does most likely not explain the very low abundance of rotifers in pond Martinodden (3) as this pond still housed populations of copepods and cladocerans. Rather, high predation could have contributed to the very low rotifer abundance in this pond. Although we did not quantify the abundance of *L. arcticus* directly, judged from the number of specimens in the samples as well as observations during the sampling, the abundance of *L. arcticus* was highest in pond Martinodden (3). *L. arcticus* is a generalist predator that consume live and dead organic matter including zooplankton (Christoffersen 2001; Jeppesen et al. 2021). In pond Martinodden (3), a larger part of the *Eurytemora rabotii* population consisted of adults as compared to pond Martinodden (1) where smaller copepodites dominated. All life cycle stages of *Eurytemora* feed on phytoplankton, but adults also eat ciliates, rotifers, and copepod nauplii (Barnes 1983). Higher predation from *L. arcticus* and adult *E. rabotii* in pond Martinodden (3) could have reduced the ciliate and rotifer abundance.

To the best of our knowledge, the impact of geese on CO_2_ and methane flux in Arctic ponds has not been investigated before. However, there are indications that high bird impact, subsidiary to other environmental drivers, may be associated with elevated methane saturation in Arctic ponds (Wei et al. 2023). Moreover, numerous studies point at a stimulating effect of nutrients and trophic status on pond emission of methane in general and CO_2_ to some extent (Davidson et al. 2015; Northington et al. 2016; Beaulieu et al. 2019;), and hence we expected an indirect, stimulating effect of bird impact on methane emission, while we expected the effect on CO_2_ to be more variable. In our study, a net CO_2_ uptake at relatively high nutrient concentrations and phytoplankton biomass most likely reflected high photosynthetic activity. Methane emission tended to be higher at higher water column TOC and water column Fe concentrations, factors that are likely affected by bird presence (Mallory et al. 2006; Duda et al. 2021). However, there was no clear relation between bird-pressure and diffusive methane flux in our data, which may indicate that other factors are mor important for the pore water nutrient concentrations. Considering the warming potential of CH_4_, ponds tended to be either net carbon sinks or small sources (pond Ureinskagen) at the time of sampling. Likely, in ponds Martinodden (3) and Muydenbukta, methane emissions were underestimated by the omission of bubble-flux measurements, despite observing ebullition in these lakes. Moreover, greenhouse gas fluxes in Arctic ponds show strong temporal variation (Laurion et al. 2010), reducing the explanatory power of these short-term sampling data. Hence, to establish the effect of bird impact on methane dynamics in Arctic freshwaters both ebullitive and diffusive fluxes over longer periods of the ice-free season need to be considered.

Moreover, in terms of the ponds’ metabolic state, our carbon flux data indicate that the ponds tended toward net autotrophy at the time of sampling, but it should be noted that due to both latitude and climate, the productive season in these high Arctic regions is short. In general, availability of light, nutrients and organic carbon, linked to landscape properties as well as seasonal and climatic features determine if freshwaters are net autotrophic or net heterotrophic systems, fuelled by terrestrial carbon sources. Lakes in arid circumpolar regions can be net autotrophic systems (Bogard et al. 2019; Ayala-Borda et al. 2024), while lakes in areas with extensive permafrost thaw receive higher organic matter loads, hence fuelling heterotrophy (Tranvik et al. 2009). Increased terrestrial or marine inputs from bird feces may shift the metabolic state of these high Arctic ponds towards heterotrophy.

Overall, our results support previous studies indicating that increasing Arctic bird populations may induce transitions between alternative ecological states (Luoto et al. 2019) or even cause regime shifts (MacDonald et al. 2015) of Arctic lentic freshwaters. Our study showed a clear split from eutrophic pond Martinodden (3) under high goose impact to hypereutrophic pond Gnålodden under the combined impact of geese and seabirds. The split was characterized by a strong increase in nutrient concentrations, especially phosphorus, causing a drastic rise in phytoplankton biomass along with a clear shift to a more species-poor phytoplankton community with dominance of cryptophytes and an increasing biomass of cyanobacteria. The hypertrophic conditions were associated with extremely low metazoanplankton species richness most likely because of a deteriorated pond environment intolerable to most metazoans. Hence, pond Gnålodden was also characterized by a trophic uncoupling where the increased organic carbon stock was unavailable for higher trophic levels due to the disappearance of the metazoanplankton and *L. arcticus*. For example, *L. arcticus* is an important food source for Arctic birds (Summerhayers and Elton 1923; Hartley and Fischer 1936). Instead, the increased availability of high-quality organic carbon of algal origin stimulates the microbial loop, as indicated by the higher biomass of mixotrophic cryptophytes, and heterotrophic flagellates and the dense population of ciliates, and prime heterotrophic microbial degradation. Trophic uncoupling may also have consequences for the availability and transfer of essential nutrients in the food web. For example, Arctic metazoan zooplankton are considered to be a richer source of essential molecules such as omega-3 fatty acids (Grosbois et al. 2022). Increasing hypertrophic conditions may therefore reduce this resource availability, for higher trophic levels. The shift in pond plankton composition and diversity related to bird impact may exemplify future scenarios where climate change and increasing Arctic bird density push Arctic ponds toward a deteriorated state of low biodiversity and altered ecological functionality.

## Supplementary Information

Below is the link to the electronic supplementary material.

Supplementary file1 (DOCX 36 KB)

## Data Availability

Data used in this paper are available on request.
