# Supplementary material for: Bird impacts on ecological structure, composition and function in Arctic ponds
Source: Polar Biol. 2025 Oct 23;48(4):111. doi: 10.1007/s00300-025-03426-1 (PMC12546552; doi:10.1007/s00300-025-03426-1)
Supplement: Supplementary file 1 — Supplementary file1 (DOCX 36 KB) [file 300_2025_3426_MOESM1_ESM.docx]

**Bird impacts on ecological structure, composition and function in Arctic ponds**

Thomas C. Jensen, Ann Kristin Schartau, Birger Skjelbred, Alexander Eiler, Maarten J. J. E. Loonen Annelies J. Veraart

**Supplementary material**

Table S1 Phytoplankton biomass (mm^3^/l) in six Svalbard ponds in 2022. A = autotrophic, H = heterotrophic, M = mixotrophic

|  |  | **Ureinskagen** | **Martinodden (1)** | **Martinodden (2)** | **Martinodden (3)** | **Gnålodden** | **Muydenbukta** |
| --- | --- | --- | --- | --- | --- | --- | --- |
| **Cyanobacteria** |  |  |  |  |  |  |  |
| Anabaena | A |  | 0,00095 | 0,01162 | 0,10970 | 0,17422 |  |
| Aphanocapsa | A |  |  | 0,00090 | 0,00163 |  |  |
| Aphanocapsa conferta | A |  |  |  |  |  | 0,03349 |
| Chroococcus | A |  |  |  |  |  | 0,00080 |
| Chroococcus dispersus | A |  |  |  |  |  | 0,00408 |
| Chroococcus prescottii | A |  | 0,02042 | 0,00327 |  |  | 0,01634 |
| Chroococcus turgidus | A |  |  |  |  |  | 0,00192 |
| Geitlerinema splendidum | A |  | 0,00028 |  | 0,00113 |  | 0,00254 |
| Gloeocapsa | A |  | 0,01258 |  |  |  |  |
| Jaaginema | A |  |  | 0,00086 | 0,00607 |  |  |
| Leptolyngbya | A | 0,00080 |  |  |  | 5,06041 |  |
| Phormidium | A |  |  |  |  | 0,06126 |  |
| Pseudanabaena limnetica | A | 0,00003 |  |  |  |  |  |
| Snowella lacustris | A | 0,01618 | 0,27751 |  |  |  |  |
| **Charophyta/Chlorophyta** |  |  |  |  |  |  |  |
| Ankistrodesmus spiralis | A |  |  | 0,00039 | 0,00408 |  |  |
| Ankyra judayi | A | 0,00817 | 0,22564 | 0,02042 |  |  |  |
| Botryococcus braunii | A | 0,00160 | 0,05216 | 0,01600 |  |  | 0,00096 |
| Chlamydomonas (l=10) | A |  |  | 0,00833 | 0,01527 |  |  |
| Chlamydomonas (l=25) | A |  |  | 0,00042 |  |  |  |
| Chlamydomonas (l=14) | A |  | 0,00184 | 0,00147 | 0,01470 | 0,11762 |  |
| Chlamydomonas (l=8) | A | 0,00327 | 0,00898 | 0,00898 | 0,04982 | 5,43372 | 0,00408 |
| Chlorophyta, spherical cells (d=10) | A |  |  |  | 0,00425 | 0,00000 |  |
| Chlorophyta, spherical cells (d=12) | A |  |  |  | 0,00070 |  | 0,00110 |
| Chlorophyta, spherical cells (d=3) | A |  |  |  | 1,79188 |  |  |
| Chlorophyta, spherical cells (d=5) | A | 0,00212 | 0,00584 | 0,00956 | 0,00319 | 0,10784 |  |
| Chlorophyta, spherical cells (d=8) | A |  | 0,00657 |  |  |  |  |
| Coelastrum microporum | A |  |  | 0,00043 |  |  |  |
| Cosmarium bioculatum | A |  |  | 0,00144 |  |  |  |
| Cosmarium botrytis | A | 0,00420 | 0,00420 |  |  |  |  |
| Cosmarium depressum | A |  |  |  |  |  | 0,00075 |
| Cosmarium laeve | A |  |  |  | 0,00150 |  | 0,00038 |
| Cosmarium punctulatum | A |  |  | 0,00490 |  |  |  |
| Cosmarium quadrum | A |  | 0,00300 |  |  |  |  |
| Cosmarium subcrenatum | A |  |  | 0,00017 |  | 0,00066 | 0,00066 |
| Cosmarium undulatum | A |  |  | 0,00160 |  |  |  |
| Elakatothrix genevensis | A |  |  |  |  |  | 0,00114 |
| Haematococcus pluvialis | A |  |  |  |  | 0,04901 |  |
| Lagerheimia ciliata | A |  |  | 0,00653 |  |  |  |
| Lanceola spatulifera | A | 0,01372 | 0,00131 | 0,01176 |  |  |  |
| Microspora | A |  |  |  |  | 0,00143 |  |
| Monactinus simplex | A |  | 0,00342 |  |  |  |  |
| Monomastix | A |  | 0,00098 | 0,00760 | 0,01555 |  |  |
| Monoraphidium arcuatum | A |  |  |  | 0,00172 |  |  |
| Monoraphidium contortum | A | 0,00049 |  |  |  |  |  |
| Monoraphidium dybowskii | A |  |  |  | 0,00139 |  | 0,00139 |
| Monoraphidium griffithii | A | 0,00082 |  |  |  |  |  |
| Oocystis | A |  | 0,01021 | 0,00163 |  |  |  |
| Oocystis marssonii | A |  |  |  |  |  | 0,00060 |
| Oocystis submarina | A | 0,00057 |  | 0,00057 |  |  | 0,00172 |
| Pandorina morum | A |  |  |  | 0,00060 |  |  |
| Paulschulzia pseudovolvox | A |  |  | 0,00257 |  |  |  |
| Planktosphaeria gelatinosa | A | 0,00075 | 0,00188 | 0,00129 | 0,00009 |  |  |
| Pseudopediastrum boryanum | A |  | 0,00240 | 0,00240 | 0,02160 |  | 0,02240 |
| Raphidocelis subcapitata | A |  | 0,00123 |  |  |  |  |
| Scenedesmus ecornis | A |  | 0,00184 | 0,00049 | 0,00490 |  |  |
| Sphaerellopsis fluviatilis | A |  | 0,00143 |  | 0,34020 | 0,43127 |  |
| Spondylosium planum | A |  |  | 0,00027 |  |  |  |
| Staurastrum alternans | A |  |  |  |  |  | 0,00060 |
| Staurastrum punctulatum | A |  | 0,00040 | 0,00120 |  |  |  |
| Tetraëdron minimum | A |  |  | 0,00041 |  |  |  |
| Tetrastrum staurogeniaeforme | A |  |  | 0,00110 |  |  |  |
| Willea rectangularis | A |  |  | 0,00019 |  |  | 0,00038 |
| Willea vilhelmii | A |  | 0,00092 |  |  |  |  |
| **Chrysophyceae/Synurophyceae** |  |  |  |  |  |  |  |
| Bitrichia chodatii | M |  |  |  |  |  | 0,00041 |
| Chromulina | M | 0,01062 | 0,09026 | 0,00584 |  |  | 0,00319 |
| Chrysococcus | M |  |  | 0,00188 |  |  | 0,00188 |
| Chrysophyceae (<7) | M | 0,02071 | 0,05468 | 0,01540 | 0,00425 |  | 0,01168 |
| Chrysophyceae (>7) | M | 0,00531 | 0,00796 | 0,01062 | 0,00796 |  | 0,00796 |
| Dinobryon sociale var. americanum | M |  |  |  |  |  | 0,02757 |
| Epipyxis alata | M |  |  |  |  |  | 0,00147 |
| Kephyrion cupuliforme | M |  |  | 0,00053 |  |  |  |
| Mallomonas | M | 0,00184 |  |  | 0,00613 |  |  |
| Mallomonas akrokomos | M | 0,00098 |  | 0,02525 |  |  |  |
| Ochromonas | M | 0,00539 | 0,16352 | 0,00898 |  |  | 0,00180 |
| Paraphysomonas | H | 0,00131 | 0,00457 | 0,00131 |  |  | 0,00131 |
| Synura, koloni | M | 0,00072 |  |  |  |  | 0,00144 |
| Uroglenopsis americana | M | 1,21598 | 0,55983 |  |  |  | 1,06237 |
| **Bacillariophyta** |  |  |  |  |  |  |  |
| Achnanthidium minutissimum | A | 0,00086 |  |  |  |  | 0,00123 |
| Cyclotella (d=10-12) | A |  |  |  | 0,00408 |  |  |
| Diatoma tenuis | A | 0,04003 | 0,00306 |  | 0,09087 |  | 0,00511 |
| Diatoma vulgaris | A |  |  | 0,00011 |  |  |  |
| Discostella stelligera | A |  |  |  |  |  | 0,01307 |
| Encyonema minutum | A | 0,00016 |  |  | 0,00041 |  |  |
| Navicula | A |  | 0,00075 |  | 0,00075 |  | 0,00400 |
| Navicula (l=15-20) | A | 0,00020 |  |  |  |  | 0,00306 |
| Nitzschia (l=100) | A |  |  |  | 0,00070 | 0,05718 |  |
| Nitzschia (l=40-50) | A | 0,00029 |  |  | 0,00357 |  |  |
| Nitzschia acicularis | A |  |  |  |  |  | 0,00071 |
| Odontidium mesodon | A |  |  |  |  |  | 0,00375 |
| Stauroneis phoenicenteron | A |  |  |  |  |  | 0,00012 |
| Ulnaria (l=110-120) | A |  |  |  |  | 0,01307 |  |
| Ulnaria (l=40-70) | A |  |  | 0,00033 |  |  | 0,00490 |
| Ulnaria (l=80-100) | A |  |  | 0,00030 |  |  | 0,00012 |
| **Dictyochophyceae** |  |  |  |  |  |  |  |
| Pseudopedinella (3 chloroplast's) | M | 0,00245 | 0,00245 | 0,00490 |  |  |  |
| **Cryptophyta** |  |  |  |  |  |  |  |
| Chroomonas | M |  |  |  |  | 40,40035 |  |
| Cryptomonas (l=20-22) | M |  |  |  | 0,30630 |  |  |
| Cryptomonas (l=24-30) | M |  |  |  |  | 0,01634 | 0,00408 |
| Cryptomonas (l=30-35) | M |  |  |  |  | 0,06616 |  |
| Katablepharis ovalis | H |  | 0,00074 |  |  |  |  |
| Plagioselmis | M |  |  |  |  | 15,99008 |  |
| Plagioselmis nannoplanctica | M | 0,05636 | 0,03553 | 0,00123 | 0,06249 |  |  |
| **Haptophyta** |  |  |  |  |  |  |  |
| Chrysochromulina parva | M | 0,00065 |  |  |  |  |  |
| **Dinophyceae** |  |  |  |  |  |  |  |
| Cystodinium | M |  |  | 0,00012 |  |  |  |
| Gymnodinium (l=14-16) | M |  |  |  |  |  | 0,00214 |
| Gymnodinium (l=30) | M | 0,00126 |  |  |  |  | 0,00126 |
| Gyrodinium helveticum | H |  |  |  |  |  | 0,00030 |
| Peridinium (l=15-17) | M |  |  |  |  |  | 0,00337 |
| **Euglenophyta** |  |  |  |  |  |  |  |
| Euglena (l=70) | M |  |  |  | 0,00105 |  | 0,00035 |
| Trachelomonas volvocinopsis | A |  |  |  |  | 6,41515 |  |
| Choanozoa |  |  |  |  |  |  |  |
| Choanoflagellates | H |  | 0,00033 | 0,00033 | 0,00098 |  |  |
| Unidentified taxa |  |  |  |  |  |  |  |
| µ-alger, Picoplankton | A | 0,00964 | 0,02548 | 0,01638 | 0,01141 | 0,19080 | 0,00363 |
| Heterotrophic flagellate (l<15) | H | 0,00245 | 0,00245 | 0,00041 | 0,00817 | 2,10712 | 0,00163 |
| Heterotrophic flagellate (l=15-20) | H | 0,00049 | 0,00214 | 0,00037 | 0,20767 | 0,06371 | 0,00061 |
| **Total phytoplankton biomass** |  | **1,43042** | **1,59975** | **0,22363** | **3,10677** | **76,75741** | **1,29491** |
